# Flexural and Impact Behaviors of Mortar Composite Including Carbon Fibers

**DOI:** 10.3390/ma15051657

**Published:** 2022-02-23

**Authors:** Md. Safiuddin, George Abdel-Sayed, Nataliya Hearn

**Affiliations:** 1 Angelo DelZotto School of Construction Management, George Brown College, 146 Kendal Avenue, Toronto, ON M5T 2T9, Canada; 2 Department of Civil Engineering, Ryerson University, 350 Victoria Street, Toronto, ON M5B 2K3, Canada; 3 Department of Civil and Environmental Engineering, University of Windsor, 401 Sunset Avenue, Windsor, ON N9B 3P4, Canada; geosayed@yahoo.com (G.A.-S.); drhearn@gmail.com (N.H.)

**Keywords:** carbon fibers, fiber content, flexural strength, flexural toughness, impact resistance, impact toughness, mortar composite

## Abstract

This study investigated the flexural and impact performances of mortar composite made with carbon fibers (MCCF). Four mortar composites (MCCF1, MCCF2, MCCF3, and MCCF4) were produced, using 1%, 2%, 3%, and 4% carbon fibers by volume, respectively. Another mortar composite without any carbon fibers (MCCF0) was prepared for its use as a control mix. The freshly mixed mortar composites were tested for inverted slump cone flow time to ensure they had an adequate workability to cast test specimens under vibration. In addition, all fresh mortar composites were examined for density and air content. The hardened mortar composites were tested for their first-crack flexural strength, ultimate flexural strength, first-crack impact resistance, and ultimate impact resistance. Moreover, the first-crack flexural toughness, ultimate flexural toughness, first-crack impact toughness, and ultimate impact toughness were determined for all hardened mortar composites. The correlations among the hardened properties of the mortar composites were also sought. Finally, the optimum fiber content was defined from the overall test results and considering the costs of the mortar composites. The test results showed that the workability and density of the fresh mortar composite decreased, whereas its air content increased due to the inclusion of carbon fibers. However, MCCF3 possessed the highest density and lowest air content among all MCCF mixes. It also had a higher workability than MCCF4. In the hardened state, the first-crack flexural strength and impact resistance, as well as the ultimate flexural strength and impact resistance of mortar composite, increased significantly with the increasing volume content of carbon fibers. In addition, the first-crack flexural toughness, ultimate flexural toughness, first-crack impact toughness, and ultimate impact toughness increased greatly with the higher volume content of carbon fibers. Strong correlations between the flexural strength and impact resistance, and between the flexural toughness and impact toughness of the mortar composites, were observed. Above all, excellent flexural strength, flexural toughness, impact resistance, and impact toughness values were observed for MCCF4 (4% carbon fibers). The 28-day ultimate flexural strength and impact resistance of MCCF4 increased by 4.6 MPa and 134 blows, respectively, as compared to MCCF0. Moreover, the 28-day ultimate flexural toughness and ultimate impact toughness values of MCCF4 were higher than that of MCCF0, by 3739.7 N-mm and 2703.3 J, respectively. However, MCCF3 (3% carbon fibers) also exhibited a good performance under flexural and impact loadings. Based on the costs of all mortar composites and their performances in both fresh and hardened states, MCCF3 was derived as the best mortar mix. This implies that 3% carbon fibers can be defined as the optimum fiber content in the context of the present study.

## 1. Introduction

Cement mortar is a popular building material that possesses good compressive strength for many applications in construction projects (e.g., load-bearing exterior masonry walls, masonry foundation walls, and masonry footings). This mortar also has good fire resistance, which makes it suitable for finishing interior walls, ceilings, and floors. However, cement mortar is inherently weak in tension, flexure, and impact. The tensile strength, flexural strength, and impact resistance of plain cement mortar are significantly low [1,2]. Therefore, this material easily cracks under such loading conditions and, hence, it demands further improvement. The cracking resistance of cement mortar can be enhanced by using discrete fibers [2,3].

Fibers are used in cement composites to improve their mechanical performances, with increases in flexural strength, toughness, and ductility at failure [4,5,6,7,8,9,10,11]. They are found to control the propagation of cracks under loading and carry a considerable amount of load after the occurrence of first crack, thus enhancing the tensile strength, flexural strength, and flexural toughness of cement composites [12,13,14,15]. Fibers also enhance the impact resistance of cement composites [16]. The most common fibers used in cement composites are steel, glass, and polypropylene [1,2]. Carbon fiber has also been incorporated into cement composites for their use in structures, particularly in the cladding and curtain walls of high-rise buildings [17,18,19,20]. It is advantageous over polypropylene, glass, and steel fibers in respect to its contribution to the finishability, weatherability, mixability, thermal resistance, and long-term chemical stability of cement composites in aggressive environments [2]. There are two main types of carbon fibers based on fiber materials, PAN (polyacrylonitrile)-based, and pitch-based carbon fibers. Mostly short pitch-based carbon fibers have been used in civil engineering applications due to its lower cost and excellent reinforcing performance in cement composites [18,20,21,22,23].

The performance of fiber-reinforced cementitious composites varies with the volume content of fibers. The increase in the volume content of fibers increases, almost linearly, the tensile strength of cement composites [24]. The higher volume content of fibers also consistently increases the flexural strength, ductility, and toughness of cement composites [25,26,27]. However, a relatively small quantity of fibers may not be adequate to improve the tensile strength, flexural strength, ductility, and toughness of a cement composite. It was noticed that cement composites experience the brittle post-peak failure if a lower volume content of fibers is used in the presence of a reactive mineral admixture, such as silica fume [2], which decreases the fracture toughness of the material [28,29]. Therefore, a higher volume content of fibers should be used in such cases to increase the ductility of cement composites. However, the increased volume content of fibers was found to deter the mixing and placement operations of fiber-reinforced cement composites by decreasing their workability [2,30]. Moreover, the cost of fibers is another factor that should be considered before its use in cement composites. It may not be economical to use a larger fiber volume content, because most fibers are expensive [31]. This is particularly vital in the case of carbon fiber, which is much costlier than the other constituents of cement composites (e.g., cement, aggregate, and chemical and mineral admixtures). Therefore, the optimum content of fibers needs to be determined to produce fiber-reinforced cement composites without any difficulties during mixing and placement, while achieving the required properties with cost-effectiveness.

Many studies were conducted to examine the effect of short pitch-based carbon fiber on the mechanical properties of cement composites in static loading conditions. Like any other fibers, carbon fiber was found to improve the tensile strength, flexural strength, and flexural toughness of cement composites [14,15,32,33]. It also increased the compressive strength of cement composites when used with a fiber content of up to 3% by volume of the composite mix [1,2,16]. Compared to static loading, less research has been conducted on the performance of carbon fiber-reinforced mortar composite under impact loading, which various structures may also undergo on many occasions. The present study emphasized the impact performance of carbon fiber-reinforced mortar composite, along with its flexural performance and key fresh properties (e.g., workability, density, and air content). In addition to the load-deflection behavior, flexural strength, and flexural toughness, the impact resistance and impact toughness of various carbon fiber-reinforced mortar composites were examined in this study. The impact resistance and impact toughness of carbon fiber-reinforced mortar composite were also correlated with its flexural strength and flexural toughness.

## 2. Research Significance

The flexural strength, flexural toughness, impact resistance, and impact toughness of the mortar composite with carbon fibers (MCCF) is important for its applications in buildings and transportation infrastructures as a new or repair material. The performances of hardened MCCF under flexure and impact loadings were investigated in this study, along with the fresh properties (workability, density, and air content). The effects of short pitch-based carbon fibers on the above-mentioned fresh and hardened properties of MCCF were examined. Under flexure and impact loadings, MCCF performed much better than plain mortar. Based on the test results and costs of all mortar composites, the best MCCF and the optimum volume content of carbon fibers were decided. Moreover, it was found that the flexural strength and flexural toughness of MCCF were strongly correlated with its impact resistance and impact toughness. These are the innovative aspects and contributions of the present study. It is expected that the overall findings of this study would be useful to produce MCCF commercially for various engineering applications.

## 3. Experimental Investigation

### 3.1. Constituent Materials of Mortar Composites

Natural river sand, normal Portland cement, silica fume, short pitch-based carbon fibers, normal tap water, and superplasticizer were used to produce different mortar composites for various tests conducted in this study. The sand was purchased from a local aggregate supplier situated in Windsor, Ontario. It was clean and free from deleterious materials, such as clay lumps and organic matter. The fineness modulus (FM), total evaporable moisture content, water absorption, and saturated surface-dry specific gravity of the sand was 1.97, 0.50%, 1.60%, and 2.60, respectively. The maximum size of the sand was 2.36 mm. The FM of the sand indicates that it was finer than the sands typically used in non-fiber mortar and concrete mixes. Pierre et al. [34] stated that the sand grading is not an important parameter for the mechanical properties of microfiber-reinforced mortars. In addition, Banthia and Genois [35] found that the sand grading has no effect on the crack growth in carbon fiber-reinforced cement composites. Indeed, the sand used in the present study did not cause any inevitable workability problems. As the mortar mixes prepared in this study contained the cement at an amount which was twice the amount of sand, the grading of sand had no significant effect on the workability of the fresh composites. Besides, the dosage of the superplasticizer was chosen in such a way so that it minimized the effect of sand’s surface fineness on the water demand and helped to obtain a workable mortar mix.

Cement manufactured by Lafarge Canada was used in the present study. It was purchased from a local construction materials supplier situated in Windsor, Ontario. The cement complied with the standard specification for Type I Portland cement and fulfilled the physical and chemical requirements given in ASTM C150/C150M [36]. The specific gravity of the cement was 3.15. The mass contents of the deleterious chemical components (e.g., MgO, SO_3_, Na_2_O, and K_2_O) of the cement were below the allowable maximum limits.

Silica fume, supplied by Master Builders Technologies Ltd., Brampton, Canada, was used in this study. It was available in a grey, fine, solid material. The silica fume conformed with the standard specification and fulfilled the physical and chemical requirements given in ASTM C1240 [37]. It was mostly composed of amorphous silica. The specific gravity of the silica fume was 2.20. The median particle size of the silica fume (0.2 μm) was 100 times smaller than that of the cement (20 μm).

**Figure 1:**
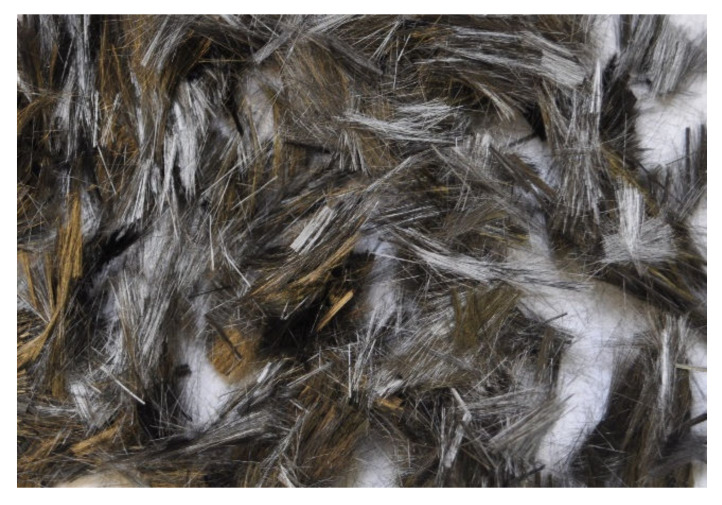
Short pitch-based carbon fibers used in various mortar composites.

Carbon fibers (CF), purchased from Mitsubishi Chemical America, Charlotte, NC, USA, were used in the present study. They were available in black chopped strands of multi-filaments (refer to Figure 1) sized with water-soluble resin. The fibers were 10 mm long and had a filament diameter of 17 μm. The specific gravity, ultimate tensile strength, and Young’s modulus of the elasticity of the carbon fibers was 1.85, 1770 MPa, and 180 GPa, respectively.

A naphthalene-based superplasticizer (SP), supplied by Master Builders Technologies Ltd., Brampton, Canada, was used in this study. The SP was available as a dark brown liquid. It did not contain any chloride and was 100% soluble in water. The pH of the SP was 8.0. It complied with the requirements of a Type F high-range water-reducing admixture given in the ASTM standard specification for chemical admixtures [38]. The specific gravity and solid content of the SP was 1.20 and 40%, respectively.

Normal tap water was used in this study for mixing and curing purposes. The quality of the water conformed to the requirements, as mentioned in the ASTM standard specification for ready-mixed concrete [39]. In general, the water quality was excellent for its use in producing the mortar composites. It did not contain any objectionable substances causing color or odor. The turbidity and total dissolved solids were 2.07 NTU and 18 mg/L, respectively.

### 3.2. Mix Proportions of Mortar Composites

Five different mortar composites, including a control mix, were designed with a water/binder (W/B) ratio of 0.35 and a sand/binder (S/B) ratio of 0.50. The control mix was the mortar composite with 0% carbon fibers (MCCF0). The other four mortar composites, namely, MCCF1, MCCF2, MCCF3, and MCCF4, were prepared with 1%, 2%, 3%, and 4% volume content of carbon fibers, respectively. Silica fume was used in all mortar composites as a 15% weight replacement of cement. Regarding the air bubbles entrapped during mixing, as a rule of thumb, 1% air content was considered for MCCF0, whereas 4% air content was assumed for MCCF1. The assumed air content was 5%, 6%, and 7% for MCCF2, MCCF3, and MCCF4, respectively. The mix proportions of the mortar composites were obtained based on the absolute volume method and were corrected considering the moisture content and absorption of the sand, as well as the water contribution of liquid SP. The corrected mix proportions of various mortar composites are given in Table 1. These mix proportions were finalized after observing the performance of the trial mixes. The acceptability of the mortar composite mixes was judged by examining their workability. An inverted slump cone flow time below 30 s was used as the acceptance criterion for the adequate workability of the fresh mortar composites, based on the findings of earlier studies [31,40,41]. The presence of carbon fibers increased the water demand to obtain workable mortar composites. Therefore, carbon fiber-reinforced mortar composites required higher SP dosages for adequate workability. The SP dosages, shown in Table 1, were decided based on the trial mixes.

### 3.3. Preparation and Testing of Fresh Mortar Composites

#### 3.3.1. Mixing of Mortar Composites

**Figure 2:**
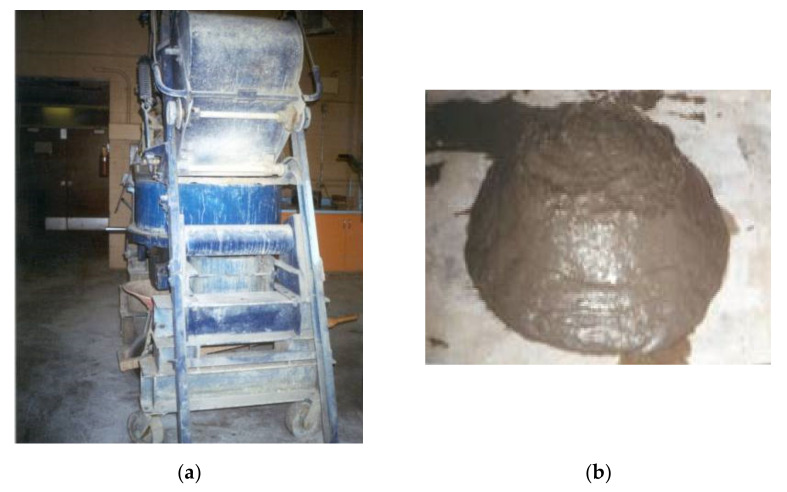
Preparation of mortar composites: (**a**) pan-type mixer used for mixing; (**b**) a portion of freshly mixed MCCF2.

The mortar composites were prepared using a pan-type mixer (100L capacity), as shown in Figure 2a. The constituent materials required for the batch volume of mortar composite were weighed before starting the mixing operation. Firstly, the sand, normal Portland cement, and silica fume were mixed for 1 min by adding half of the water content. Then, the remaining half of the water content blended with the SP dosage was poured gradually into the running mixer within 1 min, followed by an additional 1 min of mixing. Later, carbon fibers were manually spread onto the fresh mortar in the running mixer within 2 min; this step was not required in the case of MCCF0. Lastly, all mortar composites were mixed for a further 1 min. During mixing, no fiber clumping or balling in the cases of MCCF1-MCCF4 was noticed. This indicates that the carbon fibers were well-dispersed during the mixing of the mortar composites. The use of silica fume and SP was conducive to avoiding the fiber clumping or balling [42,43]. A small portion of the mortar composite, including 2% carbon fibers (MCCF2), is shown in Figure 2b.

#### 3.3.2. Workability Test

The freshly mixed mortar composites were examined for their workability with respect to inverted slump cone flow time. The flow time of the fresh mortars (MCCF0 and MCCF1-MCCF4) was measured according to ASTM C995 [44], with an exception for MCCF0. A slump cone, in its inverted position inside a wooden box, was used in this test. At first, the inverted slump cone, with a metal plate at the bottom opening, was loosely filled with the fresh mortar in one attempt. Then, the metal plate was removed and, immediately, a vibrator was used to make the mortar flow out of the inverted cone through the bottom opening. In the cases of MCCF1-MCCF4, the vibrator was moved through the cone, which was initially filled with the fresh mortar composite. No vibrator was used for MCCF0, as it was at a highly fluid state. For all mortars, the flow time was recorded by a stopwatch and used as a measure of their workability.

#### 3.3.3. Density and Air Content Test

The density and air content of the fresh mortar composites were determined in accordance with ASTM C138/C138M [45], with some exceptions for MCCF0. A cylindrical measure, specified in ASTM C29/C29M [46], was weighed to know its empty weight and was calibrated to know its volume capacity. Immediately after the completion of the mixing operation, the cylindrical measure was filled with three layers of the carbon fiber-reinforced mortar composite (e.g., MCCF1, MCCF2, MCCF3, and MCCF4). Each layer was compacted by a table vibrator. In the case of MCCF0, the cylindrical measure was filled in one attempt without any vibration because of its self-compactability. After completing the filling operation, the top surface of the mortar was leveled, and the rim of the measure was cleaned. Then the cylindrical measure, including the mortar, was weighed. The density of the mortar composite was calculated using the two weight measurements and the volume capacity of the cylindrical measure. On the other hand, the air content was determined based on the batch volume of the fresh mortar composite and the total absolute volume of its constituent materials.

### 3.4. Preparation and Testing of Hardened Mortar Composites

#### 3.4.1. Casting of Test Specimens

**Figure 3:**
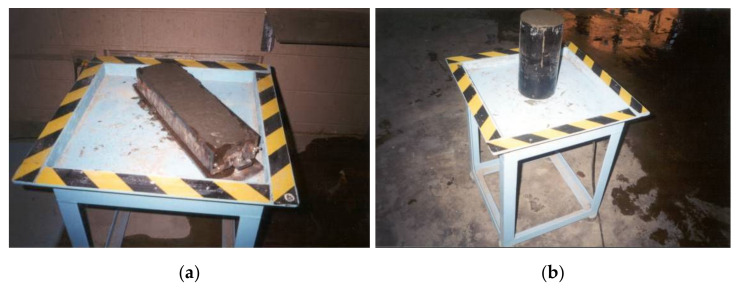
Beam (**a**) and cylinder (**b**) specimens cast for flexure and impact tests.

The beam or prism specimens of 400 mm × 75 mm × 100 mm dimensions were prepared for the flexure test. In total, 30 beam specimens were cast for 5 different mixes, considering 3 specimens at each of the 7-day and 28-day test ages. These specimens were molded in reusable cast iron molds (refer to Figure 3a). The cylinder specimens required for the impact test were prepared from Ø150 mm × 300 mm parent cylinders, which were primarily cast in single-use plastic molds (see Figure 3b). Three Ø150 mm × 62.5 mm test cylinders were obtained after cutting each Ø150 mm × 300 mm parent cylinder. In total, 10 parent cylinders were cast to obtain 30 test cylinders, which were used at the ages of 7 and 28 days. Three layers of filling were used to prepare the cylinder specimens, whereas two layers were used for the beam specimens in the cases of MCCF1, MCCF2, MCCF3, and MCCF4. A vibrating table was used for the compaction of MCCF1-MCCF4. In the case of MCCF0, while casting the specimens, the molds were filled in one attempt and no vibration was used for the compaction of the mortar because of its self-compactability. At the age of 24 h, all mortar specimens were de-molded, labelled, and then transferred to the curing tank for water curing (relative humidity: 100%, temperature: 23 ± 2 °C) until the day of testing.

#### 3.4.2. Load-Deflection Behavior and Flexure Test

**Figure 4:**
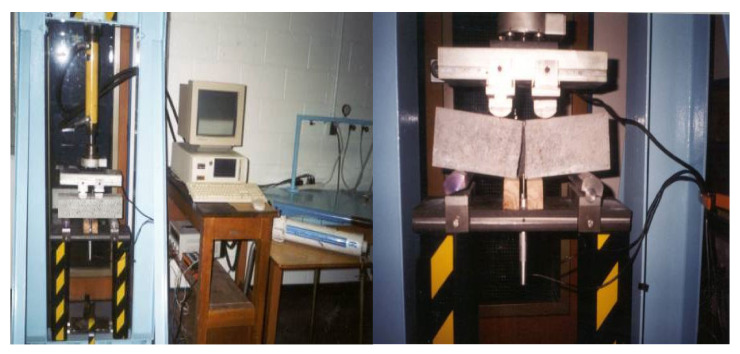
Flexure testing of mortar composite using prism or beam specimen.

The first-crack flexural strength and ultimate flexural strength of the mortar composites were determined by observing their load-deflection behavior using triplicate beam specimens (400 mm × 75 mm × 100 mm), according to ASTM C1018 [47]. The first-crack flexural toughness and ultimate flexural toughness of the mortar composites were also determined from the same test. The test set-up is shown in Figure 4. The load was applied continuously by means of a hydraulic jack. The loading system included a load-cell with a 111.2 kN (25 kips) capacity. It was connected to the data acquisition system. In addition, two transducers were used, touching the central bottom line of the specimen in a transverse direction to measure the mid-point deflection. They were also connected to the data acquisition system. Both the load-cell and the transducers were calibrated before using in the test. The first-crack load, ultimate (peak or maximum) load, first-crack deflection, and ultimate (breakpoint) deflection were noted from the gathered load-deflection data. Then Equations (1)–(4) were used to calculate the flexural strength and toughness of different mortar composites. The formulas presented in Equations (1) and (2) are available in ASTM C78 [48], whereas the formula shown in Equation (3) is based on the instruction for the first-crack toughness calculation given in ASTM C1018 [47]. The formula presented in Equation (4) was derived by the authors of this paper; it was verified using the Excel-based graphical method, which involved the counting of squares or other suitable elements of a known area:

(1) $$f_{\mathrm{fc}}=\frac{R_{\mathrm{fc}}\times \;l}{{\mathrm{bd}}^{2}}$$

(2) $$f_{u}=\frac{R_{p}\times \;l}{{\mathrm{bd}}^{2}}$$

(3) $$T_{\mathrm{fc}}=\frac{R_{\mathrm{fc}}\times {\;\Delta }_{\mathrm{fc}}}{2}$$

(4) $$T_{u}=\frac{R_{\mathrm{fc}}\times {\;\Delta }_{\mathrm{fc}}}{2}+\frac{R_{\mathrm{fc}}+{\;R}_{p}}{2}\;\left({∆}_{p}-{∆}_{fc}\right)+\frac{R_{p}+{\;R}_{b}}{2}\left({∆}_{b}-{∆}_{p}\right)$$

where b = width = 75 mm; d = height = 100 mm; l = load span (effective length) = 300 mm; f_fc_ = first-crack flexural strength (MPa); f_u_ = ultimate flexural strength (MPa); T_fc_ = first-crack toughness (N-mm); T_u_ = ultimate (total) toughness (N-mm); R_fc_ = first-crack load (N); R_b_ = breakage (breakpoint) load (N); R_p_ = ultimate (peak or maximum) load (N); Δ_b_ = breakpoint (ultimate) deflection (mm); Δ_fc_ = first-crack deflection (mm); and Δ_p_ = peak (ultimate) load deflection (mm).

#### 3.4.3. Impact Test

**Figure 5:**
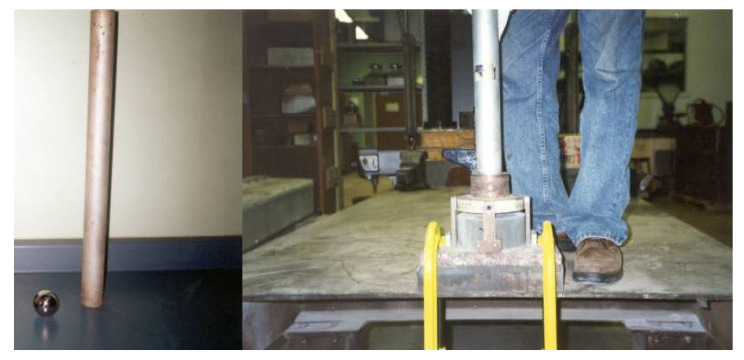
Impact test apparatus and testing of mortar composite using cylinder specimen.

The first-crack impact resistance, ultimate impact resistance, first-crack impact toughness, and ultimate impact toughness of the mortar composites were determined according to the test method given by the ACI Committee 544 [49], using triplicate cylinder specimens (Ø150 mm × 62.5 mm). The test set-up consisted of a standard 4.5 kg compaction hammer with a 457 mm drop, conforming with the ASTM specification for the use of a compacting hammer in the laboratory [50], as well as a 63.5 mm (2.5 in) diameter steel ball, and a positioning fixture to hold the cylinder specimen. The principal components of the impact test apparatus are shown in Figure 5.

The impact test was conducted by dropping the hammer repeatedly on the steel ball supported by the specimen, while observing the formation of cracks and the failure of the specimen. The numbers of blows required for the visible first crack and ultimate failure of the specimens were recorded. The amounts of impact energy required to start a visible first crack and to cause the opening of the cracks, until the failure of the specimen, were computed using Equation (5) given by the ACI Committee 544 [49]:

(5) $$I_{e/t}=\mathrm{MgHN}\;$$

where I_e/t_ = impact energy or toughness (J); N = number of blows required for fracture; M = mass of dropping hammer = 4.5 kg; g = acceleration due to gravity = 9.81 m/s^2^; and H = drop height = 0.457 m.

## 4. Test Results and Discussion

### 4.1. Fresh Properties of Mortar Composites

The inverted slump cone flow time, density, and air content results of the fresh mortar composites are shown in Table 2. The flow time varied in the range of 4–16.5 s. The flow time of the mortar without any carbon fibers (MCCF0) was 4 s. However, it greatly increased in the presence of carbon fibers, and the increase was greater for a higher volume content of fibers (see Table 2). This indicates that the workability of the mortar composite decreased due to the incorporation of carbon fibers, and this decrease was greater for a higher quantity of fibers. Such a reduction in the workability of mortar composite was mainly linked with the higher surface area of the carbon fibers, which increased the water demand for the required workability [41]. In addition, as the carbon fibers were used by mortar volume, their inclusion decreased the amounts of sand, cement, silica fume, and water in a unit volume of mortar mix, as evident in Table 1. This implies that the absolute volumes of sand, cement, silica fume, and water were decreased. However, the reduction in the total volume of cement, silica fume, and water (paste volume) was higher than the volume decrease of the sand. Consequently, it diminished the workability of carbon fiber-reinforced mortar composite. In the present study, a flow time of 30 s was used as the allowable maximum limit for acceptable workability (refer to Section 3.2). As the inclusion of carbon fibers decreased the workability of mortar composite, the SP dosage was increased to obtain a flow time below 30 s. SP acted to decrease the flow time and, thereby, facilitated the achievement of the required workability for the mortar composites prepared with carbon fibers.

The air content of the fresh mortar composites varied between 1.0 and 7.9%. The measured air content of MCCF0 was the same as its assumed air content. In contrast, the measured air content of MCCF1, MCCF2, and MCCF4 was 1–1.5% higher than their assumed air content. On the other hand, the air content of MCCF3 was 1.8% lower than its assumed air content. In general, the air content of the carbon fiber-reinforced mortar composites was significantly higher, because the presence of carbon fibers increased the quantity of entrapped air bubbles in the mortar mix. However, amongst all carbon fiber-reinforced mortar mixes (MCCF1-MCCF4), MCCF3 had the lowest air content.

The density of the fresh mortar composites varied from 1901 to 2071 kg/m^3^. In general, the density decreased when carbon fibers were incorporated into the mortar mix. The reduction in density was associated with the air bubbles entrapped in the mortar composite and was due to the lighter weight of carbon fibers, as compared to the other solid ingredients (sand, cement, and silica fume) of mortar [41]. However, MCCF3 had the highest density among all carbon fiber-reinforced mortar composites (MCCF1-MCCF4) due to its lowest entrapped air content.

### 4.2. Hardened Properties of Mortar Composites

#### 4.2.1. Load-Deflection Behavior

**Figure 6:**
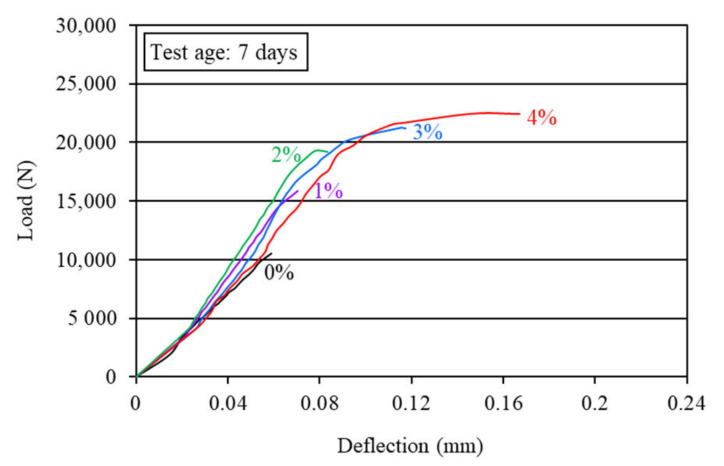
Characteristic flexural load-deflection behavior of the mortar composites at 7 days.

**Figure 7:**
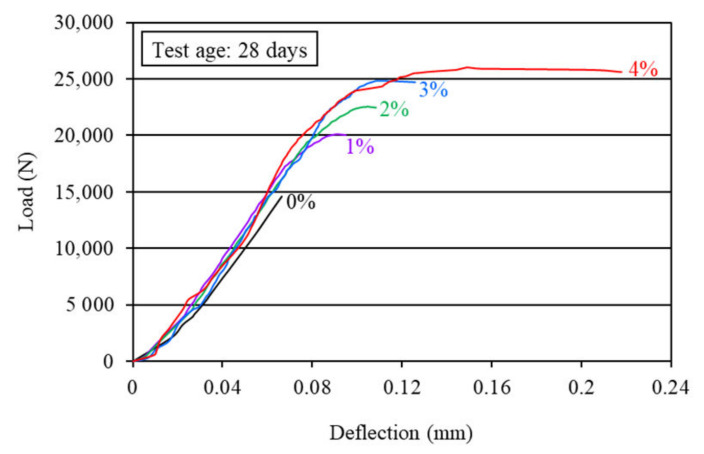
Characteristic flexural load-deflection behavior of the mortar composites at 28 days.

The water-cured hardened beam specimens (400 mm × 75 mm × 100 mm) were used to observe the load-deflection behavior of different mortar composites under flexure. The characteristic flexural load-deflection curves for various mortar composites are presented in Figure 6 and Figure 7. These two figures show that all carbon fiber-reinforced mortar composites exhibited deflection-hardening behavior. There was no post-peak deflection-softening behavior for any mix, possibly because of the presence of sand in the mortar composites. A deflection-softening behavior typically occurs in the case of the cement composites made with cement, supplementary cementitious materials, and fibers [16].

The load-carrying capacity of mortar composite increased with the increase in the carbon fiber volume content. As the load-carrying capacity increased, the beam specimens of the mortar composite made with a higher carbon fiber content exhibited a greater deflection. The breakpoint deflection increased by 40–228% for 1–4% carbon fibers. This implies that the ductility of mortar composite increased greatly with a higher volume content of carbon fibers.

The greatest load-carrying capacity and post-crack deformation at both 7 and 28 days were observed for the mortar composite that included 4% carbon fibers. Usually, the presence of silica fume increases the brittleness of mortar [2]. This was overcome by a larger fiber count (the number of fibers in a unit volume of composite) in the mortar, particularly when 3% and 4% carbon fibers were used. MCCF3 and MCCF4 exhibited larger breakpoint deflection, as evident from Figure 6 and Figure 7, thus indicating a higher ductility. This is related to the crack-bridging mechanism of fibers [51]. At 3% and 4% carbon fibers, the fiber-reinforcing area increased and, therefore, the resistance to crack propagation in MCCF3 and MCCF4 specimens was relatively high. It implies that the crack, which was initiated at the mid-span bottom of the specimens, slowed down while propagating upward. As a result, the failure of the specimens was delayed and, hence, the breakpoint deflection became greater.

#### 4.2.2. Flexural Strength

**Figure 8:**
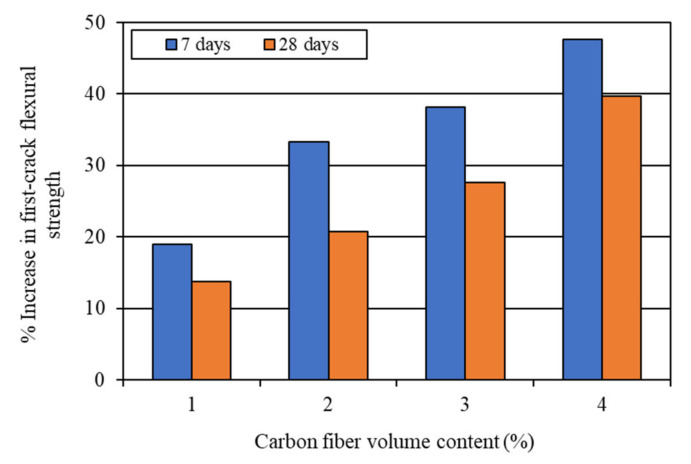
Increase in first-crack flexural strength due to the incorporation of carbon fibers.

**Figure 9:**
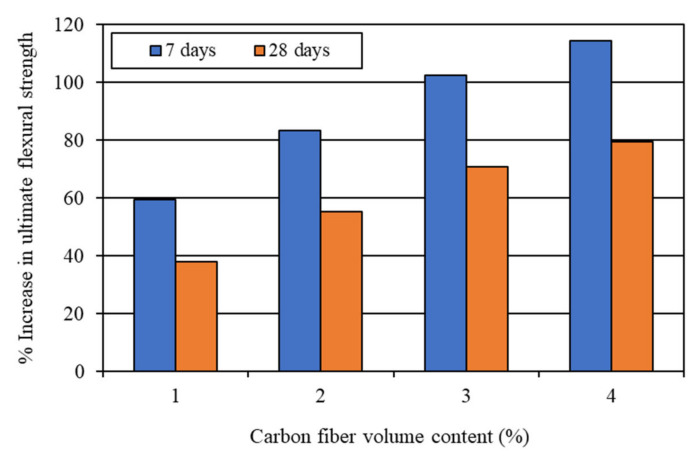
Increase in ultimate flexural strength due to the incorporation of carbon fibers.

The first-crack and ultimate flexural strengths of the mortar composites were determined at 7 and 28 days, based on the corresponding load obtained from the load-deflection curves, as shown in Figure 6 and Figure 7. The first-crack and ultimate flexural strengths were calculated using Equations (1) and (2), respectively. The average results of the first-crack and ultimate flexural strengths at the ages of 7 and 28 days are presented in Table 3. This table also shows the 7-day and 28-day first-crack and ultimate flexural toughness values, which are discussed in Section 4.2.3. The increases in the first-crack and ultimate flexural strengths for different carbon fiber volume contents are illustrated in Figure 8 and Figure 9, respectively.

The first-crack and ultimate flexural strengths of mortar composite increased at both 7 days and 28 days, with a higher volume content of carbon fibers (refer to Figure 8 and Figure 9). Akihama et al. [17], Banthia and Sheng [10], and Kim and Park [52] observed a similar trend of an increase in the flexural strength of cement composites. In the present study, the 7-day first-crack flexural strength increased by 19–47.6%, whereas the increase in the 28-day first-crack flexural strength was 13.8–39.7%. On the other hand, the 7-day ultimate flexural strength increased by 59.5–114.3%, whereas the increase in the 28-day ultimate flexural strength ranged from 37.9% to 79.3%. These increases in the first-crack and ultimate flexural strengths suggest that the incorporation of carbon fibers significantly enhanced the resistance of mortar composite to crack propagation. The mortar composite containing 4% carbon fibers (MCCF4) provided the maximum first-crack and ultimate flexural strengths at both testing ages (refer to Table 3). The mortar composite that included 3% carbon fibers (MCCF3) also exhibited greatly enhanced first-crack and ultimate flexural strengths. The first-crack flexural strength of MCCF3 increased by 38.1%, whereas its ultimate flexural strength rose by 102.4% at the age of 7 days. Furthermore, a 27.6% increase in the first-crack flexural strength and a 70.7% rise in the ultimate flexural strength were observed for MCCF3 at the age of 28 days. Such significant increases in the first-crack and ultimate flexural strengths, with the inclusion of carbon fibers, were largely credited to the increased reinforcing and bonding effects of the fibers [2,31,53]. Carbon fibers possess extremely high tensile strength (refer to Section 3.1). When they are used in cement composites, the presence of silica fume improves their bonding with the surrounding matrix [2]. In addition, they orient along the length of the beam specimens when vibration is applied for the compaction of cement composites [53]. This suggests that the carbon fibers were more effective in resisting the tensile stress at the lower portion of the specimens, below their neutral axis, thus resulting in a higher flexural strength.

#### 4.2.3. Flexural Toughness

**Figure 10:**
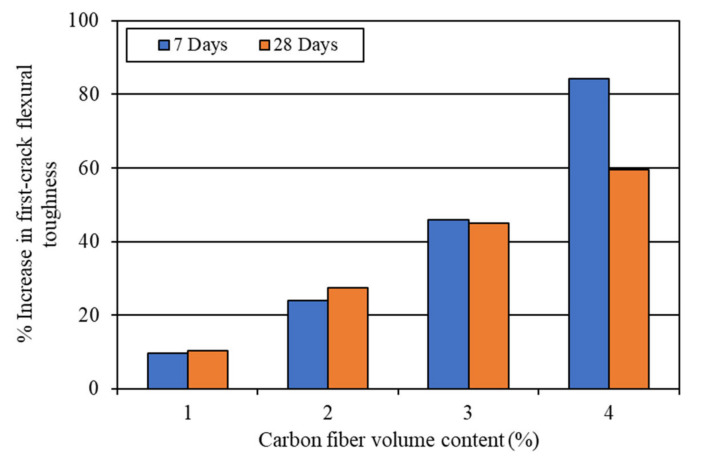
Increase in first-crack toughness due to the incorporation of carbon fibers.

**Figure 11:**
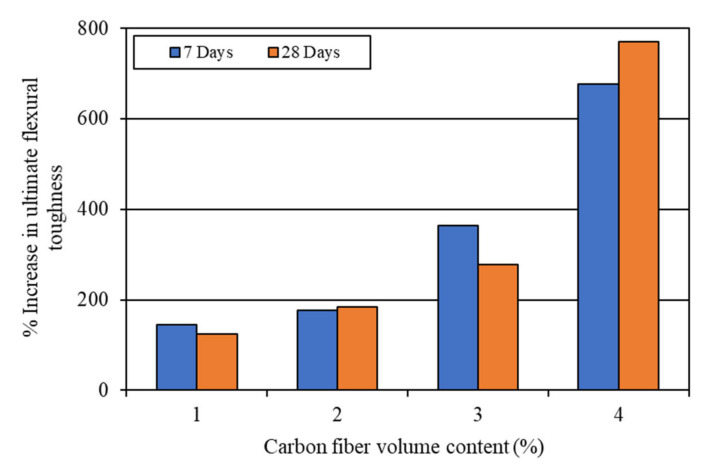
Increase in ultimate toughness due to the incorporation of carbon fibers.

The flexural toughness of the mortar composites was determined at 7 days and 28 days. The first-crack flexural toughness was computed from Equation (3), whereas the ultimate (total) flexural toughness was calculated using Equation (4), based on the corresponding load and deflection obtained from the load-deflection curves (Figure 6 and Figure 7). The average results of the first-crack and ultimate flexural toughness of the mortar composites are presented in Table 3. Both the first-crack and ultimate flexural toughness increased with a higher carbon fiber volume content. This finding is specifically illustrated in Figure 10 and Figure 11. The 7-day first-crack toughness of mortar composite increased by 9.6–84.3%, whereas the increase in the 7-day ultimate toughness was 144.7–676.4% for 1–4% carbon fiber volume contents. Furthermore, the increase in the 28-day first-crack toughness was 10.4–59.5%, while the 28-day ultimate toughness increased by 124.3–770.8% for 1–4% carbon fibers.

The mortar composite containing 4% carbon fibers (MCCF4) provided the maximum first-crack and ultimate flexural toughness at both testing ages. The first-crack flexural toughness of MCCF4 was 574.3 N-mm, whereas it was 774.0 N-mm at 28 days. In addition, the ultimate flexural toughness of MCCF4 was 2419.2 N-mm, whereas it was 4224.9 N-mm at 28 days. The greatest first-crack and ultimate toughness values of MCCF4 indicated that it was the toughest among all the composites. The mortar composite incorporating 3% carbon fibers (MCCF3) also possessed significantly high first-crack and ultimate flexural toughness values, as compared to MCCF1 and MCCF2 (refer to Table 3). The increases in the first-crack and ultimate toughness, with the incorporation of carbon fibers, were mostly due to the same reasons as discussed in Section 4.2.2 for flexural strength.

#### 4.2.4. Impact Resistance

The water-cured hardened cylinder specimens (Ø150 mm × 62.5 mm) were tested to determine the first-crack and ultimate impact resistance (with respect to the number of blows required for a visible first crack and for ultimate failure, respectively) at the ages of 7 and 28 days. The average impact test results for different mortar composites are given in Table 4. It is obvious from this table that the plain mortar took a very small number of blows for a visible first crack. It, also, did not show any considerable impact resistance beyond the first crack. Once the first crack appeared on the surface, the specimen failed with only a few additional blows. In contrast, the mortar composites incorporating carbon fibers (MCCF1-MCCF4) provided significantly improved impact resistance against the first crack and ultimate failure. In particular, MCCF3 and MCCF4, which included 3% and 4% carbon fibers, respectively, possessed very high first-crack and ultimate impact resistance. Indeed, MCCF4 provided the maximum first-crack and ultimate impact resistance, followed by MCCF3 (refer to Table 4). MCCF3 took 59 blows for a visible first crack, while it ultimately failed after 68 blows. In comparison, MCCF4 required 86 blows to produce a visible first crack, whereas 139 blows (nearly twice that of 68 blows) were needed to cause the ultimate failure of the specimen. These results show that MCCF4 was extremely resistant to impact loading. Limited studies have been conducted on the impact resistance of carbon fiber-reinforced mortar composite using the ACI drop-weight test. Ohama et al. [20] investigated the impact resistance of carbon fiber-reinforced cement, operating a similar device, and obtained comparable results. Besides, Soroushian et al. [9] carried out the ACI drop-weight test on carbon fiber-reinforced composite containing lightweight aggregates and obtained significant gains in impact resistance with increased fiber content, as observed in the present study.

#### 4.2.5. Impact Toughness

The impact toughness of the mortar composites was determined at 7 days and 28 days. The first-crack and ultimate impact toughness values were computed using Equation (5) based on the corresponding number of blows required for a visible first crack and ultimate failure (refer to Table 4). The average results of the impact toughness are shown in Table 5. It is evident from this table that the impact toughness increased greatly with the increase in the volume content of carbon fibers.

**Figure 12:**
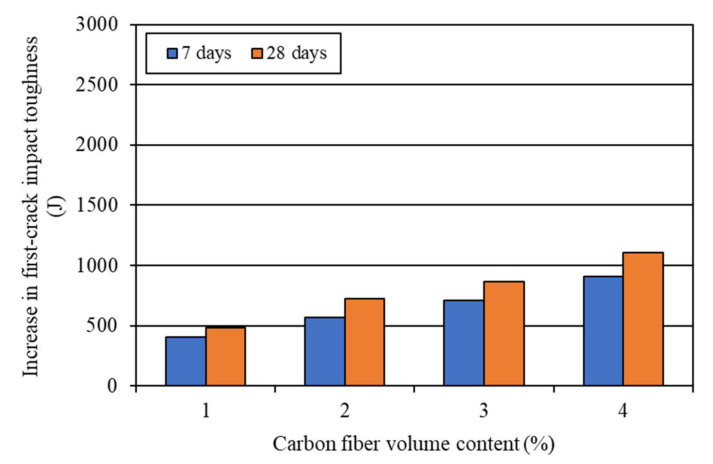
Increase in first-crack impact toughness due to the incorporation of carbon fibers.

**Figure 13:**
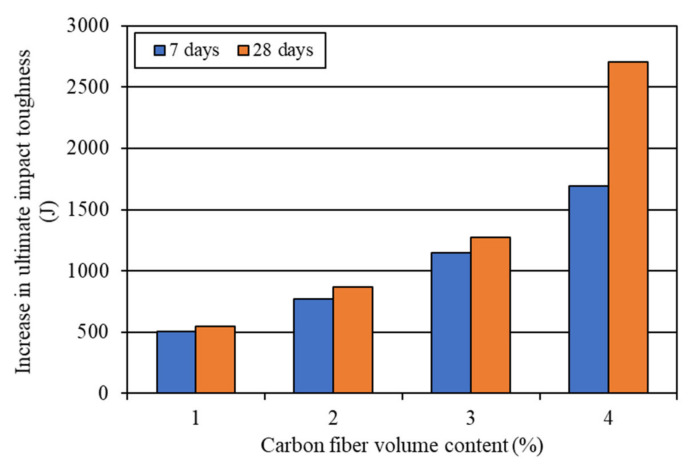
Increase in ultimate impact toughness due to the incorporation of carbon fibers.

The increases in the impact toughness of mortar composites with different carbon fiber volume contents are shown in Figure 12 and Figure 13. The 7-day first-crack impact toughness increased by 403.5–907.8 J, while the 28-day first-crack toughness increased by 484.2–1109.6 J for 1–4% carbon fibers (refer to Figure 12). On the other hand, the 7-day ultimate impact toughness increased by 504.4–1694.7 J, whereas the 28-day ultimate toughness increased by 544.7–2703.3 J for 1–4% carbon fibers (refer to Figure 13). At the age of 28 days, the ultimate toughness increased by a large amount in the case of MCCF4, which included 4% carbon fibers, as can be seen in Figure 13. The excellent improvement in impact toughness is mostly credited to the greater anchorage and better interfacial bond of carbon fibers. The presence of silica fume enhanced the anchorage and bonding of the carbon fibers in mortar composite [2,31,53].

#### 4.2.6. Correlation between Flexural Strength and Impact Resistance

**Figure 14:**
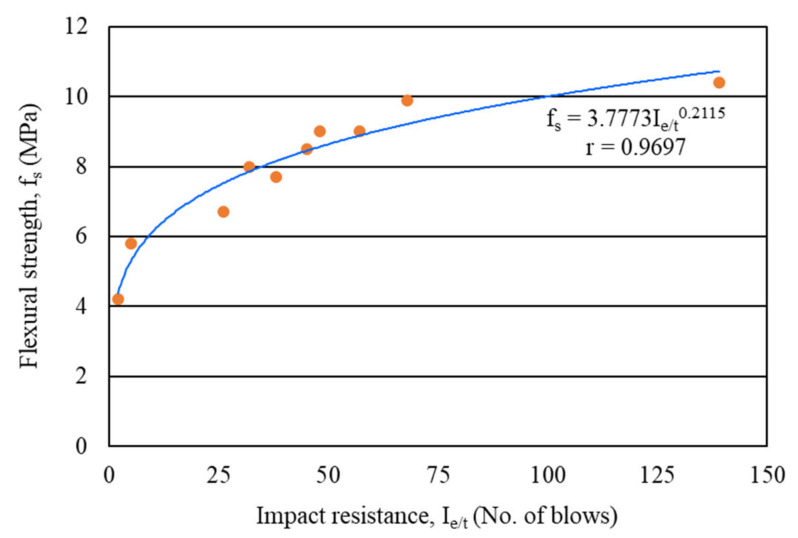
Correlation between the flexural strength and impact resistance of mortar composites.

The correlation between the flexural strength (first-crack and ultimate) and impact resistance (first-crack and ultimate) of carbon fiber-reinforced mortar composite at the age of 28 days was sought in the present study. It was found that the flexural strength of mortar composite was strongly correlated with its impact resistance. The line of best fit showed a power relationship, as observed in Figure 14. The correlation coefficient (r) was 0.9697, which indicates an excellent relationship. Such a strong relationship between the flexural strength and impact resistance of carbon fiber-reinforced mortar composite was obtained because both properties were enhanced with a higher volume content of carbon fibers. Moreover, the power relationship (Figure 14) shows that the impact resistance of mortar composite increased more pronouncedly with the volume content of carbon fibers, as compared to its flexural strength. It should also be mentioned that the ultimate impact resistance was very high for the mortar composite with 4% carbon fibers (MCCF4), as evident from Table 4. Hence, the best-fit relationship line became much milder after 75 blows.

#### 4.2.7. Correlation between Flexural Toughness and Impact Toughness

**Figure 15:**
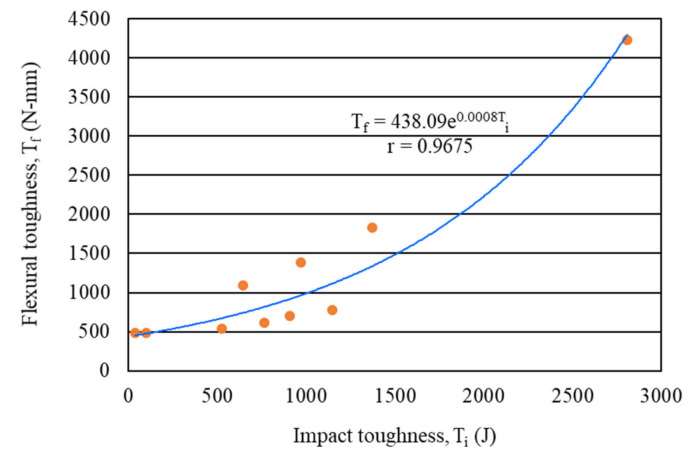
Correlation between the flexural and impact toughness of mortar composites.

The correlation between the flexural toughness (first-crack and ultimate) and impact toughness (first-crack and ultimate) of carbon fiber-reinforced mortar composite at the age of 28 days was examined in the present study. It was found that the flexural toughness of mortar composite was strongly correlated with its impact toughness. The line of best fit exhibited an exponential relationship, as evident in Figure 15. The correlation coefficient (r) was 0.9675, which suggests an excellent relationship. Such a strong relationship between the flexural toughness and impact toughness of carbon fiber-reinforced mortar composite was obtained because both properties improved with a higher volume content of carbon fibers. Furthermore, the exponential relationship (Figure 15) indicates that the flexural toughness of mortar composite increased more pronouncedly than its impact toughness for any volume content of carbon fibers. It should also be noted that, for the mortar composite with 4% carbon fibers (MCCF4), the ultimate flexural toughness increased more significantly than the ultimate impact toughness, as evident from Table 3 and Table 5. Therefore, the best-fit relationship line became much steeper after 1500 N-mm flexural toughness.

## 5. Best Mortar Composite and Optimum Fiber Content

The best carbon fiber-reinforced mortar composite was defined based on the overall performance with respect to the workability (inverted slump cone flow), density, and air content of the fresh mixes, as well as the flexural strength, impact resistance, and flexural and impact toughness of the hardened mortars. The optimum content of fibers for a mortar composite greatly depends on its mix parameters (W/B ratio, S/B ratio, etc.) and fiber characteristics (length, equivalent diameter, aspect ratio, specific surface area, reinforcement area, count, strength, toughness, etc.). For the optimization of the fiber content, the two major mix parameters, such as W/B and S/B ratios, were kept constant for all mortar composites produced in this study. Moreover, only 10 mm long carbon fibers with a 17 μm diameter were used in all mortar mixes. However, the optimum fiber content differs for different lengths and diameters of carbon fibers, as well as for a different mix composition of mortar. Therefore, the optimum content found in the present study should not be generalized.

In the context of the present study, the mortar composite with 4% carbon fibers (MCCF4) exhibited the best performance under flexural and impact loadings. However, MCCF4 possessed the lowest workability, as realized from its highest inverted slump cone flow time (refer to Table 2). Hence, MCCF4 had the highest air content and, therefore, the lowest density among all fresh carbon fiber-reinforced mortar composites. In comparison, the mortar composite with 3% carbon fibers (MCCF3) provided a higher workability than MCCF4 for its placement and compaction by vibration. The inverted slump cone flow time of MCCF3 was 48.5% lower than that of MCCF4. In addition, the freshly mixed MCCF3 had the lowest air content and highest density compared to other carbon fiber-reinforced mortar composites (refer to Table 2). In the hardened state, MCCF3 provided significantly higher flexural strength, impact resistance, and flexural and impact toughness than the other mortar composites, including those with 1% and 2% carbon fibers (refer to Table 3, Table 4 and Table 5 and Figure 8, Figure 9, Figure 10, Figure 11, Figure 12 and Figure 13). Furthermore, MCCF3 provided a reasonably high splitting tensile strength and the maximum compressive strength, which were reported in a previously published paper [53].

The cost is another factor that should be considered while optimizing the volume content of carbon fibers for mortar composite. The unit cost of carbon fibers was very high, compared to the unit costs of the other ingredients used in the mortar composites. Hence, the volume content of carbon fibers should be kept as minimum as possible without affecting the properties of mortar composite. The authors of this paper also conducted the cost–effectiveness analysis of carbon fiber-reinforced mortar composite in another study [31], comparing the workability, mechanical, and durability performances, as well as the costs of various mixes. The performance-to-cost ratio (PCR) was introduced to measure the effectiveness of a mortar composite in terms of its cost and performance. A higher PCR was associated with a greater effectiveness. It was found that MCCF3 provided the highest PCR. The cost factor (the cost of carbon fiber-reinforced mortar/the cost of plain mortar) of MCCF3 was also lower than that of MCCF4, mainly because of a lower quantity of carbon fibers [31]. Therefore, MCCF3 can be considered as the best mortar composite, based on its cost factor and performance in both fresh and hardened states. Consequently, 3% carbon fibers by volume can be decided as the optimum content in the context of the present study.

## 6. Applications of Carbon Fiber-Reinforced Mortar Composites

Carbon fiber-reinforced composite is more advantageous than other composites including polypropylene, glass, or steel fibers, due to its better finishability, higher weathering resistance, and greater chemical stability in adverse environments [2,23]. Therefore, the mortar composite with carbon fibers (MCCF) will be favorable for many applications, particularly for the manufacture of precast construction products. The high flexural strength and toughness of MCCF will be propitious for its thin sheet applications. Indeed, the cement composites made with short pitch-based carbon fibers have already been used in construction projects, mainly in the cladding and curtain walls of high-rise buildings [18,21,22,23]. It has also been used in wall and roof tiles, parapet wall panels, partition wall panels, staircase walls, roofing sheets, wall formwork, and building domes [1,2].

MCCF products will also be suitable for specific applications where dynamic impact loading is expected to happen. Dynamic impact loading can occur in many cases, such as a ship impact on a marine structure, moving vehicles on bridges, vibrating machinery, earthquakes, aircraft landings, explosive blasting, or dropped weights. In such cases, dynamic impact loading may cause severe damage to structures. As MCCF possesses high impact resistance and toughness, it would be useful to minimize the damaging effects of various impacts on structures. In addition, MCCF can be used to refurbish deteriorated concrete slab surfaces that most often occur in parking or bridge structures and industrial buildings. It can also be used as a repair or protective material for the framing elements of concrete structures (e.g., walls, columns, and beams).

## 7. Conclusions

The following conclusions are drawn from the test results of this study on carbon fiber-reinforced mortar composite:

(a) The incorporation of carbon fibers decreased the workability and increased the air content of mortar composite. Consequently, the density of mortar decreased in the presence of carbon fibers. However, the mortar composite that included 3% carbon fibers (MCCF3) had the lowest air content and the highest density among all MCCF mixes, owing to its adequate workability;
(b) The first-crack and ultimate flexural strength and toughness values of mortar composite increased with a higher volume content of carbon fibers, due to the greater resistance to crack propagation and the improved interfacial bond of the fibers with the mortar matrix;
(c) The first-crack and ultimate impact resistance and toughness values of mortar composite increased greatly with a higher volume content of carbon fibers, because the anchorage and interfacial bonding of the fibers in the mortar were greatly enhanced in the presence of silica fume;
(d) An excellent correlation was observed between the flexural strength and impact resistance, as well as between the flexural toughness and impact toughness of carbon fiber-reinforced mortar composite, because of their similar variations with the increased volume content of carbon fibers;
(e) The best performance under flexural and impact loadings was noticed for MCCF4, due to its highest volume content of carbon fibers. However, MCCF4 had the lowest workability (largest flow time) and, thus, the highest air content, creating the smallest density among all carbon fiber-reinforced mortar composites;
(f) The mortar composite that included 3% carbon fibers (MCCF3) was derived as the best mortar composite, based on its performance in both fresh and hardened states, as well as its cost factor and performance-to-cost ratio (PCR). It implies that the optimum volume content of carbon fibers was 3% in the context of the present study;
(g) Carbon fiber-reinforced mortar composite is suitable for many applications, due to its high performance in flexural and impact loading conditions.

## Figures and Tables

**Table 1:** Corrected mix proportions of various mortar composites.

Mortar Composite	Sand	Cement	Silica Fume	Water	Carbon Fibers (Vol. % of Mortar)	Superplasticizer (Wt. % of Binder *)
MCCF0	955.9	556.2	168.7	394.1	0	1
MCCF1	917.2	533.7	161.9	372.8	1	2
MCCF2	898.0	522.4	158.5	359.8	2	3
MCCF3	878.6	511.3	155.1	346.7	3	4
MCCF4	859.4	500.0	151.7	334.1	4	5

* Cement + Silica fume.

**Table 2:** Fresh properties of various mortar composites.

Type of Mortar Composite	Inverted Slump Cone Flow Time (s)	Density (kg/m^3^)	Air Content (%)
MCCF0	4	2071	1.0
MCCF1	5.5	1969	5.2
MCCF2	7	1948	6.4
MCCF3	8.5	1990	4.2
MCCF4	16.5	1901	7.9

**Table 3:** Flexural strength and toughness of various mortar composites.

Mortar Composite	First-Crack Flexural Strength (MPa)	Ultimate Flexural Strength (MPa)	First-Crack Flexural Toughness (N-mm)	Ultimate Flexural Toughness (N-mm)
**Test age: 7 days**				
MCCF0	4.2	4.2	311.6	311.6
MCCF1	5.0	6.7	341.5	762.4
MCCF2	5.6	7.7	386.5	859.8
MCCF3	5.8	8.5	455.0	1444.9
MCCF4	6.2	9.0	574.3	2419.2
**Test age: 28 days**				
MCCF0	5.8	5.8	485.2	485.2
MCCF1	6.6	8.0	535.5	1088.5
MCCF2	7.0	9.0	618.8	1381.5
MCCF3	7.4	9.9	703.0	1835.7
MCCF4	8.1	10.4	774.0	4224.9

**Table 4:** Impact resistance of various mortar composites.

Mortar Composite	Number of Blows Required for Visible First Crack (N_fc_)		Number of Blows Required for Ultimate Failure (N_uf_)	
	7 Days	28 Days	7 Days	28 Days
MCCF0	1	2	2	5
MCCF1	21	26	27	32
MCCF2	29	38	40	48
MCCF3	36	45	59	68
MCCF4	46	57	86	139

**Table 5:** Impact toughness of various mortar composites.

Mortar Composite	First-Crack Impact Toughness (J)		Ultimate Impact Toughness (J)	
	7 Days	28 Days	7 Days	28 Days
MCCF0	20.2	40.3	40.3	100.9
MCCF1	423.7	524.5	544.7	645.6
MCCF2	585.1	766.6	807.0	968.4
MCCF3	726.3	907.8	1190.3	1371.9
MCCF4	928.0	1149.9	1735.0	2804.2

## Data Availability

The data presented in this study are not publicly available.
